# Heterotroph Interactions Alter *Prochlorococcus* Transcriptome Dynamics during Extended Periods of Darkness

**DOI:** 10.1128/mSystems.00040-18

**Published:** 2018-05-29

**Authors:** Steven J. Biller, Allison Coe, Sara E. Roggensack, Sallie W. Chisholm

**Affiliations:** a Department of Civil and Environmental Engineering, Massachusetts Institute of Technology, Cambridge, Massachusetts, USA; b Department of Biology, Massachusetts Institute of Technology, Cambridge, Massachusetts, USA; Florida State University

**Keywords:** *Prochlorococcus*, interactions

## Abstract

Microbes evolve within complex ecological communities where biotic interactions impact both individual cells and the environment as a whole. Here we examine how cellular regulation in the marine cyanobacterium *Prochlorococcus* is influenced by a heterotrophic bacterium, Alteromonas macleodii, under different light conditions. We monitored the transcriptome of *Prochlorococcus*, grown either alone or in coculture, across a diel light:dark cycle and under the stress of extended darkness—a condition that cells would experience when mixed below the ocean’s euphotic zone. More *Prochlorococcus* transcripts exhibited 24-h periodic oscillations in coculture than in pure culture, both over the normal diel cycle and after the shift to extended darkness. This demonstrates that biotic interactions, and not just light, can affect timing mechanisms in *Prochlorococcus*, which lacks a self-sustaining circadian oscillator. The transcriptomes of replicate pure cultures of *Prochlorococcus* lost their synchrony within 5 h of extended darkness and reflected changes in stress responses and metabolic functions consistent with growth cessation. In contrast, when grown with *Alteromonas*, replicate *Prochlorococcus* transcriptomes tracked each other for at least 13 h in the dark and showed signs of continued biosynthetic and metabolic activity. The transcriptome patterns suggest that the heterotroph may be providing energy or essential biosynthetic substrates to *Prochlorococcus* in the form of organic compounds, sustaining this autotroph when it is deprived of solar energy. Our findings reveal conditions where mixotrophic metabolism may benefit marine cyanobacteria and highlight new impacts of community interactions on basic *Prochlorococcus* cellular processes.

**IMPORTANCE** *Prochlorococcus* is the most abundant photosynthetic organism on the planet. These cells play a central role in the physiology of surrounding heterotrophs by supplying them with fixed organic carbon. It is becoming increasingly clear, however, that interactions with heterotrophs can affect autotrophs as well. Here we show that such interactions have a marked impact on the response of *Prochlorococcus* to the stress of extended periods of darkness, as reflected in transcriptional dynamics. These data suggest that diel transcriptional rhythms within *Prochlorococcus*, which are generally considered to be strictly under the control of light quantity, quality, and timing, can also be influenced by biotic interactions. Together, these findings provide new insights into the importance of microbial interactions on *Prochlorococcus* physiology and reveal conditions where heterotroph-derived compounds may support autotrophs—contrary to the canonical autotroph-to-heterotroph trophic paradigm.

## Abstract (precis)

*Prochlorococcus* is the most abundant photosynthetic organism on the planet. These cells play a central role in the physiology of surrounding heterotrophs by supplying them with fixed organic carbon. It is becoming increasingly clear, however, that interactions with heterotrophs can affect autotrophs as well. Here we show that such interactions have a marked impact on the response of *Prochlorococcus* to the stress of extended periods of darkness, as reflected in transcriptional dynamics. These data suggest that diel transcriptional rhythms within *Prochlorococcus*, which are generally considered to be strictly under the control of light quantity, quality, and timing, can also be influenced by biotic interactions. Together, these findings provide new insights into the importance of microbial interactions on *Prochlorococcus* physiology and reveal conditions where heterotroph-derived compounds may support autotrophs—contrary to the canonical autotroph-to-heterotroph trophic paradigm.

## INTRODUCTION

Phytoplankton lie at the base of the marine food web, where their photosynthetic activity generates the organic carbon and energy on which much of the heterotrophic population depends (1). Marine autotroph-heterotroph interactions are not, however, necessarily just a one-way exchange of fixed carbon. For example, heterotrophs can release infochemicals affecting diatom cell division (2), provide essential vitamins to algae (3), scavenge reactive oxygen species (ROS) harmful to cyanobacteria (4, 5), or metabolically transform compounds released by autotrophs in ways that can impact the entire community (6, 7).

Interactions with heterotrophs are known to have diverse impacts on the marine cyanobacterium *Prochlorococcus*, the numerically dominant photosynthetic organism in the mid-latitude oligotrophic ocean (8). The presence of heterotrophs has been shown to enhance *Prochlorococcus* growth rates and resilience under optimal growth conditions (9, 10) and improve its ability to tolerate light and temperature stress (11, 12). While some of these benefits likely reflect the heterotroph’s role in reducing oxidative stress (4, 5), transcriptional studies of cyanobacterium-heterotroph interactions suggest that other unknown metabolic interactions likely contribute as well (13–17).

The physiology and ecology of *Prochlorococcus* are tightly coupled to the dependable rhythm of the daily light:dark (L:D) cycle. Growth and death are both tied to this cycle: it synchronizes cell division such that cells grow during the day and divide at night (18), and nearly all *Prochlorococcus* mortality, whether due to viral lysis, grazing, or other factors, appears to occur at night (19). Not surprisingly, most cellular transcripts display diel abundance patterns in response to the periodic influx of energy (20–25). In culture, the expression of >80% of *Prochlorococcus* MED4 protein-encoding transcripts oscillate over the diel L:D cycle (22, 23), whereas in *Synechocystis* sp. strain PCC 6803, only 30 to 40% of transcripts oscillate under similar conditions (26, 27). Diel oscillations are seen in the wild as well, where periodicity in primary production has been shown to drive transcriptional rhythms in cooccurring marine heterotrophs (24, 28). These observations highlight the fundamental importance of temporal oscillations in *Prochlorococcus*’ function to the broader marine microbial ecosystem.

The tight coupling of *Prochlorococcus* transcriptional oscillations to the light environment is particularly notable given that these cells contain a streamlined version of the KaiABC circadian oscillator system found in most other cyanobacteria (29). In Synechococcus elongatus, the KaiA and KaiB subunits regulate daily oscillations in the phosphorylation state of KaiC, which in turn regulates gene expression, cell division, and chromosome compaction dynamics in anticipation of changes in light availability (29 and 30 and references therein). These cycles in KaiC phosphorylation are largely influenced by the cellular redox state, as sensed via the quinone pools and cellular ATP/ADP ratios (31, 32). *Prochlorococcus*, in contrast, carries genes that encode only a partial circadian oscillator comprised of KaiB and KaiC; it lacks the KaiA regulator (33, 34). The resulting KaiBC oscillator cannot generate the self-sustaining diel phosphorylation rhythms observed in the complete KaiABC complex, and transcriptional oscillations quickly dampen under constant light conditions (35). This has led to the suggestion that the *Prochlorococcus* system functions not as a true clock but instead like a daily timer that is sensitive to redox changes influenced by external signals such as sunlight that serve to “reset” the KaiBC system each morning (36).

While the earth system reliably generates diel L:D cycles, the actual light environment experienced by a *Prochlorococcus* cell in the wild can be quite variable as a result of cloud cover, mixing, and internal waves. At Station ALOHA in the North Pacific Subtropical Gyre, for example, a cell living in the deep chlorophyll maximum can undergo a periodic vertical excursion of ~50 m on a daily basis, resulting in order of magnitude changes in light intensity (37, 38). Vertical water mass displacement can even plunge *Prochlorococcus* cells into total darkness, as shown by a growing number of field studies which have documented the presence of putatively intact *Prochlorococcus* cells far below the euphotic zone (11, 39–42). How, then, might cells react to such sudden changes in the regular daily L:D cycle?

We have shown that an axenic culture of *Prochlorococcus* sp. strain NATL2A (hereafter “*Prochlorococcus*”) can survive no more than ~1.5 days of darkness (11). *Prochlorococcus* can, however, withstand up to 11 days in the dark in the presence of the heterotroph Alteromonas macleodii MIT1002 (hereafter “*Alteromonas*”) with which it cooccurs in nature (43). This difference was not solely attributable to mitigation of oxidative stress by the heterotroph; other interactions appear to contribute, as demonstrated by the combinatorial impact of oxygen scavenging compounds and glucose on the ability of *Prochlorococcus* cultures to survive extended darkness (11). Here we extend these studies to the gene expression level by examining the transcriptomes of *Prochlorococcus*, grown either alone or in coculture with *Alteromonas*, as the cyanobacteria were shifted from a regular diel L:D cycle to a period of extended darkness. We sought to better understand how *Prochlorococcus* reacts to extended periods of darkness, how heterotroph interactions impact *Prochlorococcus*’ metabolism and cellular rhythms, and how these interactions might in turn affect the ability of *Prochlorococcus* to withstand darkness.

## RESULTS AND DISCUSSION

### Culture dynamics.

**FIG 1:**
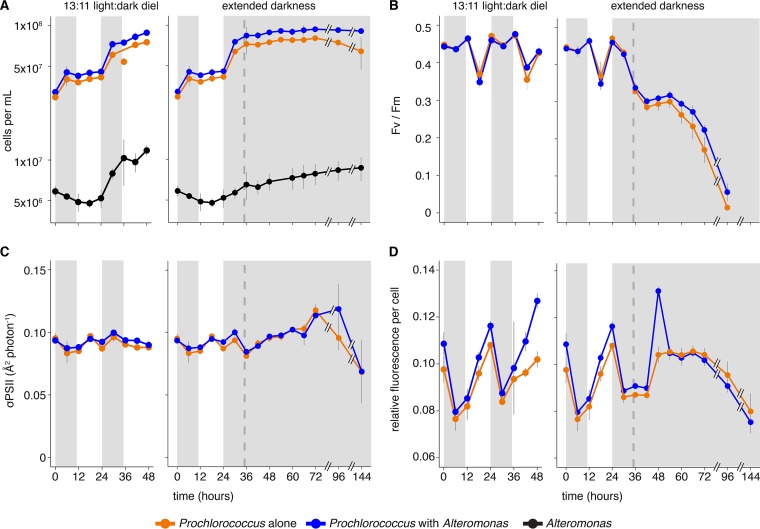
Bulk *Prochlorococcus* culture dynamics during the experiment. *Prochlorococcus* was grown either in pure culture or in coculture with *Alteromonas*. At the 24-h time point, the cultures were split and either maintained under diel light (13-h:11-h [13:11] light:dark) or moved into continuous darkness for 120 h. (A) Cell abundances. The *Prochlorococcus* exponential growth rates were 0.51 ± 0.03 day^−1^ in coculture and 0.47 ± 0.02 day^−1^ when grown alone. (B) *Prochlorococcus* photosynthetic quantum efficiency (Fv/Fm). (C) *Prochlorococcus* PSII effective absorption cross section (σPSII). (D) Relative red fluorescence per *Prochlorococcus* cell during the course of the experiment. The treatment of cells is indicated as follows: cells grown with light (white background) and cells grown in the dark (gray-shaded regions). The dashed vertical line indicates the time of the “expected” sunrise for the cultures kept in the dark. Values are means (± standard deviations [SD]) for three biological replicates. Details of statistical support for differences among cultures can be found in Table S2 in the supplemental material.

Replicate cultures of *Prochlorococcus*, either alone or in coculture with *Alteromonas*, were grown under a 13-h:11-h (13:11) L:D cycle and sampled at regular intervals for total RNA and to measure bulk culture parameters (see Table S1 in the supplemental material). The cell synchrony typical of *Prochlorococcus* when grown on L:D cycles (18, 22) is clearly visible, with division occurring primarily in the dark period (Fig. 1A). Cultures were split in half at the time of the second “sunset” and either maintained under the 13:11 diel L:D cycle or kept in the dark at the time of the expected “sunrise.” *Prochlorococcus* exponential growth rates were not significantly affected by the presence of *Alteromonas* (Fig. 1A and Table S2). When shifted into extended darkness, both axenic and cocultured *Prochlorococcus* populations began to decline in abundance after experiencing ~37 h of darkness past the expected sunrise (Fig. 1A). The *Prochlorococcus* decline was greater in the axenic cultures (20% decline from the maximum abundance) than in coculture (3% decline), indicating that the presence of the heterotroph affected cell mortality.

10.1128/mSystems.00040-18.5 TABLE S1  RNA-seq library statistics. For cocultures, the library size represents the number of read pairs obtained, and the remaining rRNAs/tRNAs include RNAs from both *Prochlorococcus* and *Alteromonas*. Mapped reads not aligned to an ORF or rRNA/tRNA represent a combination of intergenic and antisense reads, which are not considered in this study. n.a., not applicable. Download TABLE S1, XLSX file, 0.05 MB. Copyright © 2018 Biller et al. 2018 Biller et al. This content is distributed under the terms of the Creative Commons Attribution 4.0 International license.

10.1128/mSystems.00040-18.6 TABLE S2  Details of statistical analyses comparing bulk culture characterization data in Fig. 1. Download TABLE S2, XLSX file, 0.02 MB. Copyright © 2018 Biller et al. 2018 Biller et al. This content is distributed under the terms of the Creative Commons Attribution 4.0 International license.

Under the coculture conditions, *Alteromonas* cell densities were roughly 10% those of *Prochlorococcus* (Fig. 1A). Their growth roughly mirrored that of *Prochlorococcus* under diel L:D conditions, as was expected since no organic compounds were added to the medium, making heterotrophs dependent on photosynthate released from *Prochlorococcus* (16). The *Alteromonas* population continued to grow during extended darkness, though cells reached a lower maximum abundance than in the L:D culture (Fig. 1A and Table S2). Both axenic and cocultured *Prochlorococcus* remained viable during the first 35 h of extended darkness, as they were able to resume growth when placed back in the light (11) (Fig. S1).

10.1128/mSystems.00040-18.1 FIG S1  Regrowth of *Prochlorococcus* cultures following periods of extended darkness. Axenic or cocultured *Prochlorococcus* cells were placed in the dark at time zero (vertical dashed line) for the indicated number of days and then reintroduced back into a 13:11 light:dark cycle. Values represent the mean relative bulk chlorophyll fluorescence of *Prochlorococcus* cultures over time (*n* = 3 for each condition). Error bars are not shown for clarity due to slight biological variation in recovery time among replicates. Download FIG S1, PDF file, 0.1 MB. Copyright © 2018 Biller et al. 2018 Biller et al. This content is distributed under the terms of the Creative Commons Attribution 4.0 International license.

### Photosystem responses to extended darkness.

To examine the impact of coculture conditions and extended darkness on the photosynthetic capability of *Prochlorococcus*, we characterized the cultures over time using fast repetition rate fluorometry, which allows one to assess the state of a cell’s photosynthetic apparatus. The presence of *Alteromonas* had no significant impact on *Prochlorococcus*’ photosystems under a diel L:D cycle, as measured by either the photochemical conversion efficiency (variable fluorescence [Fv]/maximum fluorescence [Fm] [Fig. 1B]) or photosystem cross section area (σPSII [Fig. 1C and Table S2]). In extended darkness, however, Fv/Fm values dropped precipitously relative to cultures that remained in the L:D cycle, suggesting that cells were stressed by the lack of light energy; additionally, the decline was greater in the pure cultures than in the cocultures (Fig. 1B and Table S2). In contrast, the effective photosystem II cross section area (σPSII) increased under extended darkness relative to the L:D cycle—equally so in the pure and cocultures (Fig. 1C)—perhaps to increase the chances of capturing light by expanding the antenna complex. Average fluorescence per cell was slightly greater in the cocultures than in the axenic cultures during the L:D cycle and remained so for the first few hours of extended darkness (Fig. 1D and Table S2). This suggests that cocultured *Prochlorococcus* maintained higher levels of chlorophyll synthesis than the axenic cells, perhaps reflecting heterotroph shading and an increased ability to synthesize (or maintain) chlorophyll under extended darkness. In short, though there were minor differences, the bulk physiological behaviors of the two sets of cultures were similar for at least a day under the stress of extended darkness. Major differences were apparent, however, at the transcriptome level.

### Changes in replicate behavior during extended darkness.

**FIG 2:**
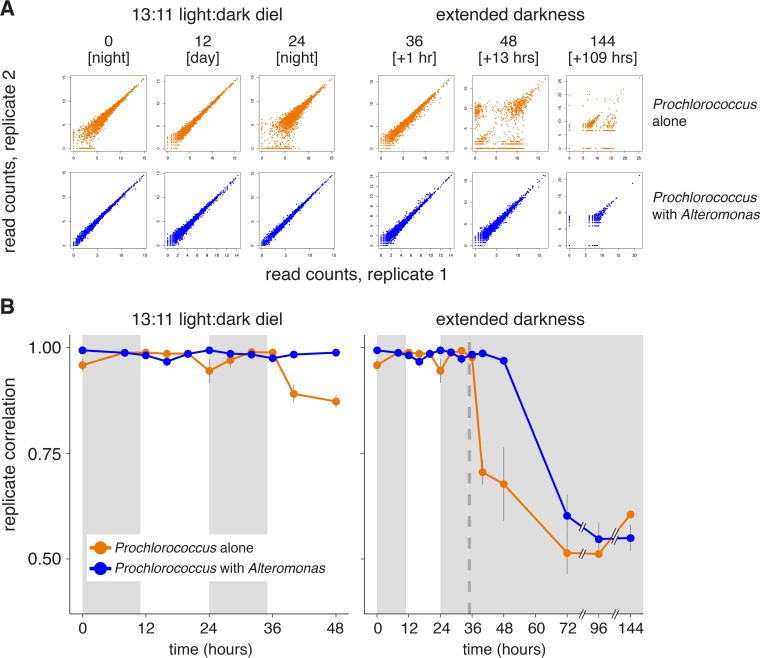
Impact of coculture on *Prochlorococcus* transcriptome replicate concordance. (A) Scatterplots comparing relative transcript abundance for each *Prochlorococcus* ORF (individual dots) between two replicates at the indicated time points for the axenic (orange) or cocultures (blue) (see also Fig. S2 for all pairwise combinations). Read counts were log transformed. Values in brackets indicate either the time of day during the day or the number of hours under extended darkness. (B) Spearman correlation of transcriptome replicates (such as those shown in panel A) at each time point. Values indicate the means (± SD) of all possible pairwise combinations of the three replicates. The dashed vertical line indicates the time of “expected” sunrise for cultures kept in the dark.

We were surprised to find that one of the strongest signals emerging from the transcriptome data was a rapid decline in concordance among the three axenic biological replicates in extended darkness compared to those on the diel L:D cycle. The replicate divergence was clear after ~5 h in extended darkness and increased thereafter (Fig. 2). This was not the case for the coculture transcriptomes, where replication held tight at least 13 h longer than the axenic cells (Fig. 2B and Fig. S2). We interpret this result as a biological response, given that the large decrease in replicate correlation coincided with the change in light conditions, was greater in the axenic extended-darkness culture than in the parallel axenic L:D controls, and was not seen in coculture libraries from the same time points (which were prepared simultaneously; see Materials and Methods). The transcriptome sequencing (RNA-seq) library preparation procedures used, including rRNA depletion methods, have been shown to robustly preserve mRNA relative abundances (44, 45), and we do not find a relationship between replicate correlation and rRNA removal efficiency (Table S1; Spearman correlation rho *P* = 0.59).

10.1128/mSystems.00040-18.2 FIG S2  Concordance among replicate transcriptomes. The relative abundance of transcripts for each ORF (black dots), compared among all three replicate transcriptomes, under the 13:11 diel light:dark cycle (A) and extended darkness (B). The replicates compared are indicated at the top of each column. Read counts are log transformed. Values in brackets indicate the time of day (A) or hours under extended darkness (B). See Fig. 1 for the corresponding culture growth behavior at these time points. Download FIG S2, PDF file, 1.3 MB. Copyright © 2018 Biller et al. 2018 Biller et al. This content is distributed under the terms of the Creative Commons Attribution 4.0 International license.

Transcriptome correlations decreased slightly each day around sunset (Fig. 2B, orange line, 0- and 24-h time points) in the axenic cultures that remained under a diel L:D cycle, but not in the cocultures. This raises the possibility that the ability of pure *Prochlorococcus* to maintain tight control on gene expression is affected during the metabolic transition from photosynthesis to the use of internal energy stores at night and implicates heterotrophs as the source of required resource(s) during this time. The response might also be attributable, at least in part, to stresses imposed by an accumulation of reactive oxygen species during the day (46). We hypothesize that the slight drop in replicate correlation among the axenic L:D control cultures during the second light period compared to the first light period (Fig. 2B) could reflect an increased contribution of reactive oxygen species to transcriptome variation arising from the greater numbers of cells present later in the time course.

Since RNA-seq data represent the average transcript abundance from a large population of cells subjected to essentially identical conditions, it seems unlikely that the differences among replicate transcriptomes represent uniform shifts in expression strategies within each culture bottle. The simplest explanation for the observed patterns is that they arose from stochastic differences in global gene expression that emerged among individuals or subpopulations of cells within the extended-darkness replicates, likely driven by the lack of available energy or required precursors (see below). The vast majority of the axenic *Prochlorococcus* transcriptome was affected by the shifts in replicate concordance, with 70% of transcripts exhibiting high dispersion during the first 13 h of extended darkness compared to only 2% in *Prochlorococcus* cocultures (Fig. S3). Such variation also points to potential limitations inherent in *Prochlorococcus*’ transcriptional architecture, which has relatively few protein regulatory factors and likely relies instead on RNA-based regulatory strategies (47), which might not have been sufficiently resilient or robust to this particular stress condition. As our methods were designed to focus only on mRNA changes, future studies will be needed to understand the role that antisense RNAs, noncoding RNAs, or other forms of RNA-based regulation (48) may play in mediating *Prochlorococcus* stress responses.

10.1128/mSystems.00040-18.3 FIG S3  Distribution of per-gene dispersion estimates for the four culture conditions. Dispersion (variance) estimates were calculated using the normalized relative abundance of each transcript at the 36-, 40-, and 48-h time points (the series of samples that correspond to the “expected” dusk-to-dawn period for the “extended-darkness” cultures), using default DESeq2 functions across all triplicate samples (see Materials and Methods). Larger dispersion values indicate greater variance among replicates. Distributions for the four conditions were all significantly different from one another (Wilcoxon rank sum test with Benjamini-Hochberg multiple-testing correction, *P* < 2 × 10^−16^), except between the *Prochlorococcus* with *Alteromonas* cocultures grown under diel 13:11 light:dark versus extended-darkness conditions (*P* = 0.096). The numbers of transcripts with dispersion of >2 under the different conditions were as follows: *Prochlorococcus* alone (L:D diel), 0; *Prochlorococcus* alone (extended darkness), 1,569; *Prochlorococcus* with *Alteromonas* (L:D diel), 40; *Prochlorococcus* with *Alteromonas* (extended darkness), 54. Download FIG S3, PDF file, 0.1 MB. Copyright © 2018 Biller et al. 2018 Biller et al. This content is distributed under the terms of the Creative Commons Attribution 4.0 International license.

In other biological systems, stochastic gene expression can reflect the action of a “bet-hedging” mechanism, in which resulting phenotypic diversity within a population improves the chances that some portion of the cells might survive a particular stress (49). In contrast, here we found that the axenic cells, which had the most transcriptional variation, were less able to resume growth upon reexposure to light than cocultured cells (Fig. S1). Though this correlation is also potentially complicated by additional limitations in energy or metabolic intermediates (discussed below), in the end, the cultures with the most variation—the axenic cultures—were less, not more, resilient to stress, arguing against any “bet-hedging” operating successfully in this case.

### Overall transcriptome shifts.

**FIG 3:**
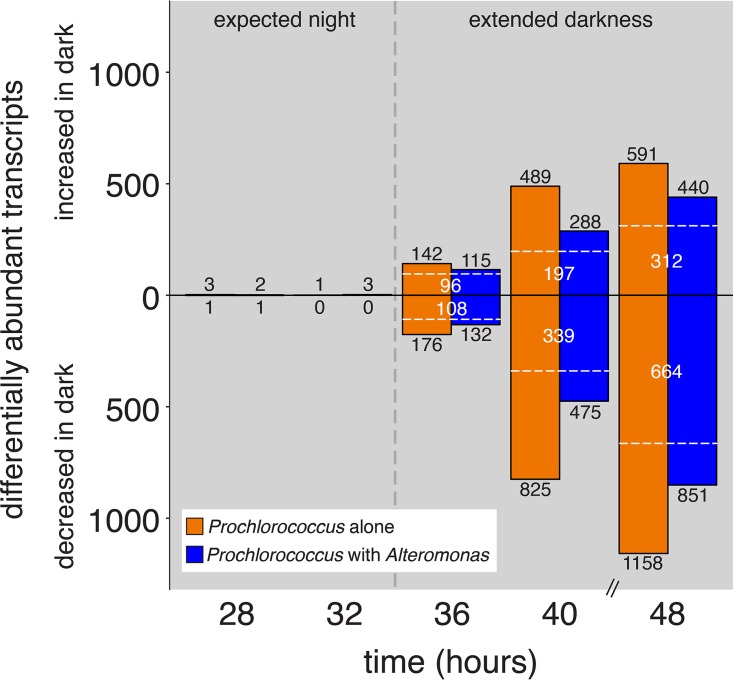
Dynamics of *Prochlorococcus* transcriptome changes during the transition from the expected dark period into extended darkness. Values depict the number of significantly differentially abundant transcripts at each time point in either axenic (orange) or cocultured (blue) *Prochlorococcus* cultures which were kept in the dark compared to the corresponding control cultures that remained under a 13:11 diel L:D cycle. Under the unperturbed light cycle, sunrise would have occurred at the 35-h time point (indicated with a black vertical dashed line); the 28- and 32-h time points represent times during the “expected night” period, prior to the shift into extended darkness, and serve as a control. Transcripts significantly enriched in the “extended darkness” cultures are represented by bars above the center line, with depleted transcripts below the center line; the bars at 28 and 32 h are too small to see at this scale. The number of enriched or depleted transcripts are indicated in black numbers; transcripts common to both axenic and cocultures are noted by white dashed lines and numbers.

After just 1 h of extra darkness, transcripts representing 14% of all 2,198 *Prochlorococcus* open reading frames (ORFs) were significantly differentially abundant in the axenic cells compared to the controls—increasing to nearly 80% of ORFs after 13 h in the dark (Fig. 3). The cocultured *Prochlorococcus* behaved similarly, though fewer ORFs exhibited significant changes compared to controls. Some transcripts changed similarly in both cocultured and axenic cultures, but there were also many differences unique to both the axenic and coculture conditions (Fig. 3). Overall, more transcripts significantly decreased in relative abundance than increased under extended darkness relative to the control, with some transcripts depleted to below detectable levels in any replicate (Fig. 3 and Fig. S4). Though the total number of detected ORFs declined over time in all dark-exposed cultures, the decline was delayed by at least 8 h in cocultured cells compared to the axenic cultures. These shifts in overall transcriptome content and complexity were not simply attributable to differences in sequencing depth (Table S1) and are consistent with the hypothesis that cocultured *Prochlorococcus* cells were less limited for either energy or some resource compared with axenic cells in the dark.

10.1128/mSystems.00040-18.4 FIG S4  Fraction of total *Prochlorococcus* ORFs detected in the transcriptome data sets. Values indicate the proportions of all 2,198 *Prochlorococcus* NATL2A ORFs with at least one mapped RNA-seq transcript in any of the biological replicates (i.e., the union of all three replicate data sets) for a given experimental condition at each time point sampled. The top bar shows the diel light pattern (light [white] and dark [black]). The gray vertical line indicates the time of the “expected” sunrise for the extended-darkness cultures. Download FIG S4, PDF file, 0.1 MB. Copyright © 2018 Biller et al. 2018 Biller et al. This content is distributed under the terms of the Creative Commons Attribution 4.0 International license.

### Transcriptomic response of axenic cultures to extended darkness.

Our more detailed analysis of the axenic transcriptomes focused on statistically significant differences apparent primarily during the first 5 h past the missed sunrise, while cells were still viable (Fig. S1) and before the replicates had maximally diverged (Fig. 2B); transcripts present after days in the dark are discussed below. The bulk of the transcriptional response was consistent with the cessation of growth in the dark (Fig. 1A). Cells were depleted in transcripts encoding a number of biosynthetic pathways, NAD metabolism genes, and ATP synthase subunits relative to cells that experienced sunrise on schedule (Table 1 and Table S3), thus implying that axenic *Prochlorococcus* cultures generally decreased their metabolic activity under extended darkness. The activity that continued appeared to be focused largely on recovering energy and/or nutrients. For example, cells likely catabolized cellular components in the dark, as enzymes in degradative pathways were enriched compared with the control (Table 2). Some activity also appeared directed toward collecting and utilizing photons, given that the transcriptomes were enriched for genes involved in the light reactions of photosynthesis (including photosystem I [PSI] and PSII components and enzymes involved in light-harvesting compound synthesis), adenylate kinase, and respiratory functions (Table 2 and Table S3). This is consistent with the observed increase in photosystem cross section area under extended darkness (Fig. 1C).

**TABLE 1:** *Prochlorococcus* metabolic pathways with transcripts that were consistently depleted during the first 5 h of extended darkness^a^

Condition and pathway^b^	Transcripts in pathway exhibiting significant differential depletion	*P* value^c^
Depleted in both axenic cultures and coculture		
Chemoautotrophic energy metabolism	*ctaC*, *ctaD*, *ctaE*, *spt*	2.1E−02
CO_2_ fixation	*cbbA*, *gapA*, *glxK*, *pgk*, *rbcL*, *rbcS*, *tktA*	1.2E−02
Generation of precursor metabolites and energy	*aspC*, *cbbA*, *ctaC*, *ctaD*, *ctaE*, *eno*, *gap2*, *gapA*, *glk*, *gltA*, *glxK*, *gpmI*, *gnd*, *mqo*, *ndhE*, *ndhI*, *petH*, *pgk pgl*, *ppc*, *pta*, *rbcS*, *rbcL*, *spt*, *tktA*, *ubiA*, *zwf*, PMN2A_0452, PMN2A_1511	9.8E−03
Glycogen degradation	*glgP*, *malQ*, *pgm*	8.8E−02
Nucleoside and nucleotide biosynthesis	*apt*, *atpA*, *atpB*, *atpC*, *atpD*, *atpE*, *atpF*, *atpG*, *atpH*, *carA*, *cca*, *codA*, *dcd*, *glxK*, *guaA*, *guaB*, *ndk*, *nrdA*, *purE*, *purH*, *purK*, *purL*, *purM*, *purS*, *purT*, *pyrB*, *pyrC*, *pyrD*, *queA*, *thyX*, *tmk*, *rnpA*, *upp*, PMN2A_0984, PMN2A_1284	1.6E−03
Oxidative pentose phosphate pathway	*gnd*, *pgl*, *zwf*, *tktA*	6.0E−03

Depleted only in axenic cultures		
Cob(II)yrinate *a*,*c*-diamide biosynthesis	*cbiD*, *cobB*, *cobI*, *cobK*, *cobM*, *cobN*, *cysG*	2.0E−02
l-Isoleucine biosynthesis	*aspC*, *ilvA*, *ilvB ilvD*, *ilvE*, *lysC thrA*, PMN2A_1138	3.0E−02
Homoserine and methionine biosynthesis	*aspC*, *lysC*, *met3*, *met17*, *metA*, *metB*, *thrA*, PMN2A_1269, PMN2A_1748	1.0E−02
NAD metabolism	*nadB*, *nadD*, *nadE*, *pntA*, *pntA-2*, *pntB*, *ppnK*	2.0E−02
Other amino acid biosynthesis	*argC*, *lysC*, *met17*, *thrA*, PMN2A_1832	8.0E−02
Polymer/polysaccharide degradation	*glgP*, *glk*, *malQ*, *pgm*	9.0E−02
Pyrimidine nucleotide *de novo* biosynthesis	*carA*, *ndk*, *nrdA*, *pyrB*, *pyrC*, *pyrD*, *thyX*, *tmk*	8.0E−02
*S*-Adenosyl-l-methionine biosynthesis	*lysC*, *metA*, *metB*, *thrA*, PMN2A_1269, PMN2A_1748	7.0E−02

Depleted only in coculture		
Aerobic respiration	*ctaC*, *ctaD*, *ctaE*, *ndhE*, *ndhI*	9.0E−02
Amine/polyamine/urea degradation	*ureA*, *ureB*, *ureC*	5.0E−02
Carbohydrate biosynthesis	*cbbA*, *gap3*, *gapA*, *glgC*, *malQ*, *pgk*, *pgm*, *rbcL*, *rbcS*, *tktA*	5.0E−02
Degradation/utilization/assimilation	*carA*, *cbbA*, *gcvP*, *gapA*, *glmU*, *glnA*, *malQ*, *met3*, *pgk*, *pgm*, *rbcL*, *rbcS*, *speA*, *tktA*, *ureA*, *ureB*, *ureC*	8.0E−02
Glycolysis	*cbbA*, *eno*, *gap3*, *pgk*	6.0E−02
Pentose phosphate pathway	*gnd*, *pgl*, *tktA*, *zwf*	2.0E−02
Pyrimidine nucleobase salvage II	*codA*, *upp*	3.0E−02
Superpathway of serine and glycine biosynthesis I	*glyA*, *serA*	9.0E−02

a *Prochlorococcus* metabolic pathways with transcripts that were consistently depleted during the first 5 h of extended darkness (36- and 40-h time points) relative to the controls grown on a 13:11 diel light:dark cycle. Additional details on these and other significantly depleted transcripts can be found in Table S3 in the supplemental material.

b Pathway definitions are from BioCyc (75).

c *P* value indicates the result of a Fisher significance test (PathwayTools).

**TABLE 2:** *Prochlorococcus* metabolic pathways with transcripts that were consistently enriched during the first 5 h of extended darkness^a^

Condition and pathway^b^	Transcript(s) in pathway exhibiting significant differential enrichment	*P* value^c^
Enriched in both axenic cultures and coculture		
Acetyl-CoA biosynthesis, carboxylate degradation	*acoA*, *lpd*, *pdhB*	1.3E−02
Degradation/utilization/assimilation	*acoA*, *deoC*, *gldA*, *glpX*, *gltB*, *lpd*, *murQ*, *pdhB*, *pgi*, *prkB*, *rpi*, *tpiA*, *truB*, PMN2A_1709, PMN2A_1584	6.6E−02
Generation of precursor metabolites and energy	*acnB*, *acoA*, *glpX*, *icd*, *lpd*, *ndhH*, *petC*, *petD*, *pgi*, *pdhB*, *prkB*, *psaD*, *psbB*, *pykF*, *rpi*, *tal*, *tpiA*, PMN2A_1709	1.7E−02
Secondary metabolite biosynthesis	*dxs*, *gldA*, *ispE*, *ispG*, *lytB*, *pykF*, *tpiA*	4.6E−02
Sugar degradation	*deoC*, *pgi*, PMN2A_1709	3.0E−03

Enriched only in axenic cultures		
Alcohol biosynthesis degradation, glycerol degradation	*gldA*, *pykF*, *tpiA*	1.5E−02
CO_2_ fixation	*glpX*, *prkB*, *rpi*, *tpiA*	1.2E−02
Fatty acid and lipid biosynthesis	*accA*, *accC*, *fabI*, *lpxC*, PMN2A_1785	1.5E−02
Fermentation	*acnB*, *icd*, *pdhB*, *pykF*, PMN2A_1709	1.0E−03
Photosynthesis	*glpX*, *tpiA*, *prkB*, *rpi*, *psaD*, *psbB*, *petD*	2.0E−03
Respiration	*acnB*, *icd*, *lpd*, *pdhB*, *pykF*	4.0E−02
RubisCO shunt	*prkB*, *pykF*, *rpi*	5.5E−02
Unsaturated fatty acid biosynthesis	*fabI*, PMN2A_1785	3.6E−02

Enriched only in coculture		
Adenine and adenosine salvage	*apt*	6.8E−02
Lipoate salvage	*lplA*	6.8E−02
Proteinogenic amino acid degradation	*lpd*, *gltB*, PMN2A_1709	1.0E−01
Purine nucleotide degradation	*truB*	6.8E−02
Terpenoid biosynthesis	*dxs*, *ispG*, *ispE*, *lytB*	6.0E−03
Tetrapyrrole biosynthesis	*hemB*, *hemD*	7.5E−02

a *Prochlorococcus* metabolic pathways with transcripts that were consistently enriched during the first 5 h of extended darkness (36- and 40-h time points) relative to the controls grown on a 13:11 diel light:dark cycle. Additional details on these and other significantly enriched transcripts can be found in Table S3.

b Pathway definitions are from BioCyc (75). Acetyl-CoA, acetyl-coenzyme A.

c *P* value indicates the result of a Fisher significance test (PathwayTools).

10.1128/mSystems.00040-18.7 TABLE S3  All *Prochlorococcus* transcripts found to be significantly differentially abundant at any time point between 36 and 48 h (1 to 13 h in extended darkness). Values indicate log_2_ fold change in cultures experiencing extended darkness versus 13:11 light:dark controls. Statistically significantly different transcript abundances (as determined by DESeq2 at a corrected *P* value of <0.1) are indicated by three asterisks. Download TABLE S3, XLSX file, 0.3 MB. Copyright © 2018 Biller et al. 2018 Biller et al. This content is distributed under the terms of the Creative Commons Attribution 4.0 International license.

During a typical dark period, cyanobacteria convert their glycogen stores into glucose, which is then degraded via the oxidative pentose phosphate pathway (50). *Prochlorococcus* does not appear to carry glycogen stores sufficient to sustain itself past the expected sunrise, as reflected in the depletion of transcripts for genes involved in glycogen degradation, such as *glgP*, as well as the oxidative pentose phosphate pathway and the late steps of glycolysis (Table 1 and Table S3). Alternatively, some cyanobacteria are known to instead use fermentation pathways to recover energy under dark anoxic conditions (50). *Prochlorococcus* excretes a variety of organic compounds such as glycolate and acetate (51), and potentially citrate and pyruvate (52), that could serve as terminal electron acceptors for fermentation. That *Prochlorococcus* turned to fermentation during extended darkness is suggested by the enrichment of multiple fermentation-associated transcripts in the axenic cultures (Table 2). We also found enrichment of both pyruvate kinase (*pykF*), which generates pyruvate and ATP from phosphoenolpyruvate, as well as the proposed pyruvate efflux transporter (PMN2A_0294) during the dark (Table 2 and Table S3). While this suggests the cells were able to recover energy via fermentation, it was not sufficient to sustain cells for long in the dark, since the cultures could not regrow after more than a day of extended darkness (Fig. S1) and exhibited clear transcriptional signals of ceasing growth within hours.

Besides the shifts in growth, biosynthesis, and energy recovery, signs of general cellular stress were also apparent in the transcriptomes. For instance, the axenic *Prochlorococcus* transcriptomes were enriched in transcripts for a variety of common stress-responsive genes such as *groES*, *clpB*, and *dnaJ* within the first hour of extended darkness (Table S3). We also found activation of the stringent response, a conserved bacterial pathway that downregulates cellular growth in response to a variety of stress conditions and is also known to contribute to dark adaptation in the cyanobacterium Synechococcus elongatus PCC 7942 (53). Forty-one of the 43 S. elongatus genes regulated in the dark via the stringent response and with a homolog in *Prochlorococcus* also changed similarly in the axenic cultures (53) (Table S3), indicating that the stringent response likely contributes to *Prochlorococcus*’ reaction to extended darkness. In addition, transcripts encoding CCRG-1 (NATL2_11351), a gene previously identified as being induced under coculture conditions (15), were enriched during extended darkness in both the axenic and cocultures (Table S3), suggesting that CCRG-1 may be responsive to other types of stress or physiological conditions even in axenic cells.

### Distinct coculture responses to extended darkness.

While some features of the axenic and cocultured *Prochlorococcus* transcriptomes responded similarly to extended darkness, there were also clear differences (Fig. 3 and Tables 1 and 2). In our earlier work (11), we postulated that coculture with a heterotroph stabilizes *Prochlorococcus* during extended darkness through a combination of (i) mitigation of reactive oxygen stress and (ii) supplying organic carbon for mixotrophic metabolism (54–58). Consistent with the first factor, we found that transcripts for a number of genes involved in DNA repair, such as *recA*, *ruvB*, *mutS*, and *radA*, were enriched only in the axenic cells, not in cocultures, under extended darkness (Table S3). With regard to the second factor, a number of signals in the transcriptome data support the idea of mixotrophic metabolism occurring within the cocultured cells. For example, many of the increases in transcript abundance for photosystem subunits (e.g., *psbA*, *psbB*, *psaA*, and *psaB*) seen in the axenic cultures did not occur in the cocultures (Table 2 and Table S3), suggesting that the latter may have had additional energy sources. Further, cocultured, but not axenic, *Prochlorococcus* cells were enriched in transcripts encoding organic compound degradation/salvage pathways, such as those for amino acids, that could be used by the cell to process organic substrates (Table 2). Although the oxidative pentose phosphate pathway was depleted under extended darkness in all cultures (Table 1), organic molecules could have been metabolized via the Entner-Doudoroff (ED) pathway, which has been proposed to be the primary means by which *Prochlorococcus* oxidizes glucose under mixotrophic conditions (59). Consistent with this, transcripts for the key enzyme in the ED pathway, Eda (2-keto-3-deoxygluconate-6-phosphate aldolase), were enriched in coculture and consistently depleted in axenic cultures (Table S3). Though the behavior of other transcripts in the ED pathway did not behave uniformly in extended darkness (e.g., we also found dark enrichment of *gdh*, no change in *gk*, and depletion of *edd*; Table S3), the differences seen between the cocultured and axenic cells suggest that glycolytic metabolism was different in the presence of *Alteromonas*, likely reflecting increased processing of organic molecules.

Transcripts involved in some amino acid and nucleotide biosynthetic pathways were depleted under extended darkness in the axenic cells, but not in the cocultures (Table 1), suggesting that the latter maintained greater biosynthetic potential in the dark. The enrichment of salvage pathways in the cocultures indicates that the unknown compound(s) we propose were supplied by *Alteromonas* could also have been required biosynthetic intermediates. For example, most nucleotide biosynthesis occurs at night in *Prochlorococcus* (22), and if nucleotide/nucleic acid pools made during the normal dark period were utilized by the cell for energy or other purposes during the initial hours of extended darkness, axenic cells may not have had sufficient precursors left to make new nucleotides. This would limit the pool available for gene expression and, from there, biosynthesis—perhaps contributing to the observed variability in transcriptional regulation in the dark (Fig. 2 and Fig. S2 and S3). In contrast, if *Alteromonas* supplied compounds needed for new gene expression and/or biosynthesis in *Prochlorococcus*, this would also help explain their ability to sustain metabolism longer.

Further, the transcriptome data show that dark activation of the stringent response, which was seen in the axenic cultures, did not occur similarly in cocultures. Out of the putatively stringent-response-regulated transcripts in *Prochlorococcus*, 39% (16/41) responded differently to extended darkness in axenic versus cocultures (Table S3). Notably, cocultures did not exhibit any enrichment for *hpf*, which encodes a stringent-response-regulated ribosome hibernation-promoting factor proposed to play a key role in S. elongatus dark survival (53). Though nutrient starvation is only one possible upstream trigger for the stringent response, this result is consistent with the hypothesis that *Prochlorococcus* acquired organic carbon from *Alteromonas* in the cocultures.

To better understand these putative metabolic exchanges in the cocultures, we examined differences in transporter expression in *Prochlorococcus* during extended darkness. Transcripts of 24 transporter-related genes significantly decreased in relative abundance during the first 5 h of extended darkness in axenic cultures; of these, 14, including a number of ABC transporters of unknown substrate, a putative dipeptide permease, and the Pro1404 transporter, which can take up glucose (54), were similarly depleted in the presence of *Alteromonas* (Table S3). The axenic transcriptomes were also specifically enriched in both a phosphate transporter and an iron transporter (Table S3), suggesting additional nutrient requirements for these cells perhaps associated with the increased expression of photosystem components. The fact that transcript levels of at least 10 transporters were relatively depleted in axenic but not cocultured *Prochlorococcus* transcriptomes raises the possibility that cocultures could have maintained a relatively higher uptake potential. Also of note is that transcripts for two putative efflux transporters proposed to excrete citrate (PMN2A_1303) and glycolate (PMN2A_1891) in *Prochlorococcus* (52) were significantly depleted in axenic cultures but remained relatively unchanged in coculture (Table S3). This pattern could indicate that citrate, glycolate, or a related overflow metabolite from *Prochlorococcus* may have been involved in the metabolic conversation with *Alteromonas*. While it is clear that any organic uptake that occurred was insufficient for growth of *Prochlorococcus* in the dark (Fig. 1A), interactions with *Alteromonas* clearly affected the *Prochlorococcus* transcriptome and allowed the cyanobacteria to persist for much longer in the dark than they could on their own (Fig. S1).

As there was no organic carbon source added to the media, essentially all of the organic carbon in these cultures was produced photosynthetically by *Prochlorococcus*. Thus, the stark differences between the responses of the axenic and cocultures imply that whatever compound or compounds sustain *Prochlorococcus* in extended darkness are not substances that it released into the culture and then took back up when needed (what we call the “pantry” hypothesis). Rather, some amount of processing by the heterotroph was likely required to convert photosynthate into a form that *Prochlorococcus* itself could utilize (which we call the “premastication” hypothesis)—an expansion, of sorts, of the canonical autotroph-heterotroph paradigm. Heterotroph-driven nutrient recycling has been demonstrated in *Synechococcus-Roseobacter* cocultures, where *Roseobacter*-mediated remineralization of dissolved organic matter was found to contribute to the long-term (weeks to months) stability of cocultures (7). Though this could be occurring to some degree in our experiment, the transcriptome and physiology data suggest that, under these shorter time scales and stress conditions, the cycling of organic materials is likely to be more important to *Prochlorococcus* survival than removal of accumulated organic matter or other toxic by-products.

### Impact of light and coculture on transcript periodicity.

Since the *Prochlorococcus* KaiBC timer cannot sustain free-running 24-h periodic oscillations, this system would not be expected to function in continuous darkness. Thus, some of the transcriptome responses described above could be related to the impacts of extended darkness on periodic regulation. Consistent with this, we found that transcripts encoding 69% of all *Prochlorococcus* proteins exhibited 24-h periodicity in the axenic L:D cultures, while only 6% did so through the first 13 h of extended darkness (Table 3 and Table S4A). This loss of periodicity was expected, but we were surprised to find that its features were affected by the presence of *Alteromonas*: more *Prochlorococcus* transcripts retained their periodicity in cocultures versus axenic cultures, both under extended darkness and even during the normal diel L:D cycle (Table 3). In turn, transcripts from 13% of all *Alteromonas* genes exhibited 24-h periodicity when grown on a diel L:D cycle in coculture with *Prochlorococcus*—consistent with field observations showing heterotroph transcript oscillations under natural L:D cycles (24). *Alteromonas* transcript periodicity essentially vanished in the extended-darkness cultures, supporting the idea that this behavior was driven by the metabolism of *Prochlorococcus* (Table 3 and Table S4B). Though the analysis of *Prochlorococcus* periodicity could be affected by shifts in replicate concordance, the fact that extended darkness led to a substantial decline in oscillating transcripts in coculture even though replicate correlations did not differ substantially over the first 48 h of the experiment (Fig. 2) argues against this being the primary driver.

**TABLE 3:** Periodic transcriptional dynamics during a consistent diel light:dark cycle and extended darkness

Organism (growth condition)	No. of transcripts (%) with 24-h periodicity^a^ under:	
	13:11 light:dark cycle	Extended darkness
*Prochlorococcus* (grown alone)	1,515 (69)	127 (6)
*Prochlorococcus* (grown with *Alteromonas*)	1,877 (85)	555 (25)
*Alteromonas* (grown with *Prochlorococcus*)	530 (13)	2 (0.05)

a Values are the number of ORFs whose relative transcript abundance oscillated with significant 24-h periodicity across the first 48 h of the experiment and their percentage of the 2,198 total annotated *Prochlorococcus* genes or 4,213 total annotated *Alteromonas* genes.

10.1128/mSystems.00040-18.8 TABLE S4  *Prochlorococcus* (A) or *Alteromonas* (B) genes whose transcripts were expressed with 24-h periodicity. Transcripts for a gene that were significantly periodic under the indicated experimental condition are indicated by a “Y” (for yes). See Materials and Methods for analytical details. Download TABLE S4, XLSX file, 0.2 MB. Copyright © 2018 Biller et al. 2018 Biller et al. This content is distributed under the terms of the Creative Commons Attribution 4.0 International license.

**FIG 4:**
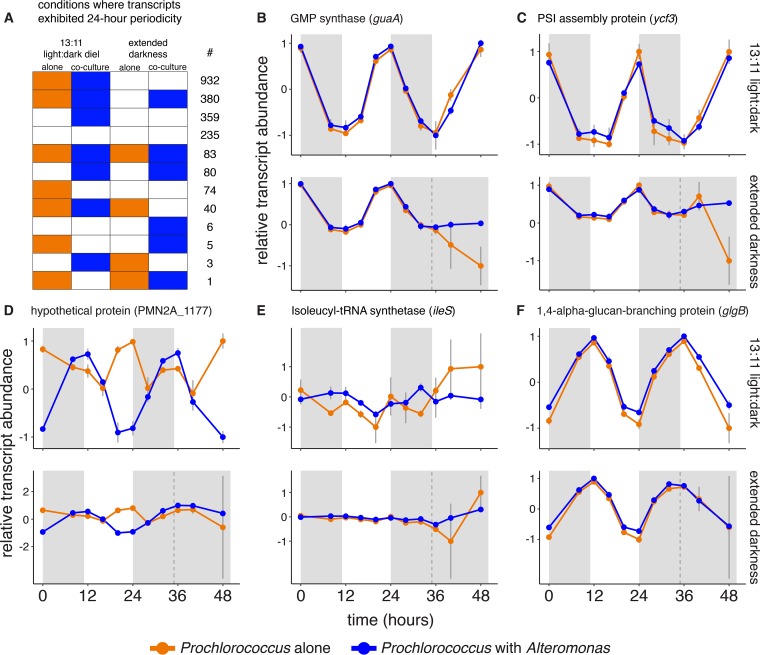
Impact of coculture on *Prochlorococcus* periodic transcriptional oscillation patterns during normal diel light and extended darkness. (A) Number of *Prochlorococcus* transcripts exhibiting significant 24-h periodicity under different experimental conditions. Colored boxes indicate cultures where transcript abundance was periodic; white boxes represent conditions where transcripts were not significantly periodic. (B to F) Representative transcript abundance profiles for the five most frequently observed groups shown in panel A. Plots show normalized relative transcript abundance under the 13:11 diel L:D cycle (top plot) or after the shift into extended darkness (bottom plot). The treatment of cells is indicated as follows: cells grown with light (white background) and cells grown in the dark (gray-shaded regions). The dashed vertical line indicates the time of the “expected” sunrise for the cultures kept in the dark. Values are means (± SD) for three biological replicates.

Within these general trends in periodic behavior, we found that subsets of the *Prochlorococcus* transcriptome exhibited periodic oscillations under different combinations of culture conditions (Fig. 4 and Table S4A). The largest group of *Prochlorococcus* transcripts (42% of all protein-encoding genes) showed 24-h periodicity in both axenic and cocultures under diel L:D conditions but did not continue to oscillate under extended darkness (Fig. 4A and B and Table S4A). Other transcripts maintained periodic oscillations in different sets of culture conditions, including those that oscillated under all conditions or only as a function of coculture or that did not exhibit periodicity under any conditions (see Fig. 4B to F for representative examples). Looking more closely at the set of transcripts that continued oscillating under extended darkness in cocultured but not in axenic cells, we find transcripts associated with a variety of metabolic pathways, including the Calvin cycle, glycolysis, fatty acid biosynthesis, glycogen metabolism, and photosynthesis (Table S4A). This further indicates that the presence of the heterotroph may help *Prochlorococcus* survive the stress of extended darkness by contributing to the maintenance of regular metabolic functionality. It is not yet clear what may have caused any particular transcript to keep oscillating under one condition and not another, though metabolic limitations and multiple other factors could have contributed to this result, as discussed below.

The differences in periodic expression between axenic and cocultured *Prochlorococcus*—and particularly the observation that this occurred even under an unperturbed diel L:D cycle—lead us to hypothesize that metabolic interactions with *Alteromonas* can influence cellular regulation in *Prochlorococcus*. The influence of *Alteromonas* on *Prochlorococcus* periodic expression may have occurred through one or more mechanisms that combined to directly or indirectly influence the cellular redox state, which serves as a key input to the circadian clock system responsible for regulating diel transcriptional oscillations (29). One possibility is that respiration of organic carbon compounds cross-fed from *Alteromonas* influenced the redox state of the *Prochlorococcus* quinone pool. Another potential mechanism for this is through the malate uptake pathway, which modulates redox balance by transferring electrons directly into the quinone pool via the malate:quinone oxidoreductase (Mqo). This pathway has previously been proposed to facilitate *Prochlorococcus* uptake of organic carbon at night (52). While transcripts for *mqo* and the proposed uptake transporter (PMN2A_1755) were significantly depleted in axenic cultures during extended darkness, they were either unchanged or less depleted under coculture conditions (Table S3), consistent with a role for heterotroph-supplied organic carbon in mitigating the impact of dark stress.

The presence of reactive oxygen species can also influence cellular redox balance (60), raising the possibility that *Alteromonas*-driven ROS detoxification (4, 5) could have indirectly affected the redox-sensitive Kai system. Consistent with this hypothesis, we observed that under extended darkness, *Prochlorococcus* transcriptomes were enriched for transcripts encoding two putative Na^+^/H^+^ antiporters (*kefB* and *nhaP*; Table S3), which play important roles in modulating cellular pH homeostasis (61). Transcripts for both genes were enriched to a lesser degree in the cocultures than the axenic cells during extended darkness, pointing toward further differences in cellular states that could have influenced the *Prochlorococcus* timing system. We also propose that the *Prochlorococcus-*driven oscillations within *Alteromonas* cells may have led to temporally coupled metabolic feedback loops within the community, which ultimately reinforced *Prochlorococcus*’ own transcriptional rhythms in coculture. Since circadian rhythms contribute to the fitness of cyanobacteria (62), this raises the possibility that some of the benefits *Prochlorococcus* derives from coculture (9, 10), even under reproducible L:D cycles, could arise from the improved synchrony of daily gene expression oscillations.

### *Prochlorococcus* transcriptomes after multiple days in the dark.

The analysis thus far has focused on the first 13 h of extended darkness, but *Prochlorococcus* cells swept below the euphotic zone might remain in the dark for days to months or longer. Although axenic cells were not able to resume growth following more than 1 day in the dark, transcripts from 100 different *Prochlorococcus* genes were found consistently in both axenic and cocultures between ~1.5 and 4.5 days of extended darkness, at which point we stopped sampling (Fig. S1 and S4 and Tables S1 and S5). This indicates that at least some fraction of the cells remained intact (as was previously observed [11]) and retained some physiological activity. What is not clear is whether these transcripts are products of transcriptional activity in the dark or are long-lived transcripts generated earlier; however, given that the half-lives of *Prochlorococcus* transcripts are typically on the order of minutes under normal growth conditions (63), we think the latter is unlikely. This raises the possibility that a portion of cells maintained a basal level of transcriptional activity in extended darkness or entered into a state of dormancy where the mRNAs were stabilized in some way.

10.1128/mSystems.00040-18.9 TABLE S5  *Prochlorococcus* transcripts consistently present in the late time points of extended-darkness cultures. Transcripts for a gene that fall in the category listed at the top of the column are indicated by an “X”. The 72- to 144-h time points represent 37 to 109 h past the expected sunrise. The mapped transcript read counts were >0 in all three time point samples. Download TABLE S5, XLSX file, 0.05 MB. Copyright © 2018 Biller et al. 2018 Biller et al. This content is distributed under the terms of the Creative Commons Attribution 4.0 International license.

The transcripts observed under the longer dark-exposure time frame encoded, among other proteins, RNA polymerase subunits, ATP synthase components, multiple photosystem I and II genes, an iron starvation-induced chlorophyll binding protein, multiple ribosomal proteins, RubisCO large and small chains, enzymes involved in central carbon metabolism, and chaperones (Table S5). Additional sets of transcripts were found specifically in either the cocultures or axenic cultures, indicating that coculture conditions continued to exert an influence even after days in the dark (Table S5). Though it is not clear how these transcripts were distributed among individual cells or subpopulations, they could represent the minimal core functionality needed to “reactivate” cells after reintroduction to the light.

### *Alteromonas* transcriptomic response.

While the focus of this study was on the response of *Prochlorococcus*, the extended-darkness condition also had a clear impact on the transcriptome of *Alteromonas*. About 20% of the transcripts of all of its ORFs were differentially abundant at each time point examined during extended darkness (Table S6A). We found changes in the transcripts encoding central metabolism and biosynthetic pathways, as would be expected given the differences in its growth dynamics (Fig. 1A), as well as shifts in the relative abundance of transcripts for outer membrane proteins and transporters (Table S6B). These observations are consistent with the general hypothesis that *Alteromonas*’ metabolism was active and could have either directly (i.e., from internal stores) or indirectly (through processing of *Prochlorococcus* photosynthate) released organic carbon useful to *Prochlorococcus* in the dark (11). Since *Alteromonas* growth was dependent on *Prochlorococcus* photosynthate, we could not compare these results to pure heterotroph cultures to determine which responses were specific to the presence of *Prochlorococcus*.

10.1128/mSystems.00040-18.10 TABLE S6  (A) Number of significantly differentially abundant Alteromonas macleodii MIT1002 transcripts between cultures remaining in the diel light:dark cycle versus extended darkness. All differences were statistically significant as determined by DESeq2 and a corrected *P* value of <0.1. Percentages reflect the fraction of all 4,213 annotated MIT1002 protein-encoding genes. (B) Alteromonas macleodii MIT1002 transcripts consistently differentially abundant following 1 to 5 h of extended darkness. Values indicate log_2_ fold change in extended darkness versus diel controls. All differences were statistically significant as determined by DESeq2 and a corrected *P* value of <0.1. Download TABLE S6, XLSX file, 0.04 MB. Copyright © 2018 Biller et al. 2018 Biller et al. This content is distributed under the terms of the Creative Commons Attribution 4.0 International license.

### Conclusions.

Our findings add to a growing body of literature describing how the physiology of *Prochlorococcus* is coupled to that of the surrounding community (4, 24, 52, 64). A *Prochlorococcus* cell’s response to the stress of light energy deprivation is clearly markedly different when grown alone versus with another microorganism, and though microbial interactions could not completely compensate for the metabolic consequences of light deprivation, *Prochlorococcus* clearly benefitted from heterotroph interactions under the stress of extended darkness. These data suggest that *Prochlorococcus* cells, which evolved within a generally reproducible light environment, do not accumulate internal stores in excess of what they need during the expected nighttime; in the rare instances where they experience the stress of extended darkness, they are critically dependent on community interactions to facilitate their survival. Our results also point to the importance of community metabolic interactions in one of the most fundamental features of cellular regulation in *Prochlorococcus*—the coordination of transcriptional oscillations with the daily L:D cycle—and raise further questions concerning the role that community interactions may have played in shaping the evolution of the timing mechanism in *Prochlorococcus*. Further, while it is well-appreciated that the transcriptomes (and hence meta-metabolism) of microbial communities in the wild are coordinated by the metabolic fluxes from phytoplankton to heterotrophs (24), our results suggest that fluxes from heterotrophs to phytoplankton (7, 52) merit further investigation. It will be particularly interesting to examine the contributions of metabolic interactions to *Prochlorococcus* rhythms in the euphotic zone, as well as the coupling and feedback between autotroph and heterotroph transcriptional oscillators. To this end, future efforts should work toward identifying the compounds exchanged between *Alteromonas* and *Prochlorococcus* and quantifying their fluxes in both directions.

## MATERIALS AND METHODS

### Culturing and sampling.

Axenic *Prochlorococcus* NATL2A cells were grown in natural seawater-based Pro99 medium containing 0.2-µm-filtered Sargasso Sea water, amended with Pro99 nutrients (N, P, and trace metals) prepared as previously described (65). Alteromonas macleodii strain MIT1002 (43) was maintained in ProMM medium (Pro99 medium, as above, plus lactate, pyruvate, glycerol, acetate, and Va vitamins) (66). At the onset of the experiment (4 days prior to sample collection), A. macleodii MIT1002 was spun down and washed twice in Pro99 medium to minimize carryover of trace organic compounds prior to being added to the *Prochlorococcus* NATL2A cultures. The selected concentration of A. macleodii MIT1002 added was previously shown not to inhibit or enhance NATL2A growth rates during exponential growth phase (16).

Three 4-liter bottles of axenic *Prochlorococcus* NATL2A and three bottles containing *Prochlorococcus* NATL2A plus A. macleodii MIT1002 were grown at 24°C in a 13-h:11-h (13:11) light:dark (L:D)  cycle with simulated dawn and dusk (22), at 55 µmol photons m^−2^ s^−1^, and bubbled with air. Transcriptome sampling began 4 days after the cultures had been inoculated, at which point cells had reached mid-exponential growth phase. After the first 24 h of sampling, the cultures in the 4-liter bottles were split in half and placed in 2-liter bottles (also bubbled with air). Half of these cultures remained in the 13:11 L:D incubator, and the other half were moved into a dark incubator at 24°C. Cultures were monitored daily by bulk chlorophyll fluorescence (Synergy 2; BioTek, Winooski, VT).

The cultures were sampled across two complete diel cycles at 0, 8, 12, 16, 20, 24, 28, 32, 36, 40, and 48 h. Beyond this, additional samples of the cultures that remained in the dark were collected at the 72-, 96-, and 144-h time points. Transcriptome samples were obtained by removing 5 ml of culture and placing it immediately into 15 ml of 4°C RNALater. This sample was incubated at 4°C for 1 to 3 days, filtered onto 25-mm 0.2-µm Supor filters (Pall, Port Washington, NY), and finally frozen and stored at −80°C until library preparation. All dark sampling and measurements were done in green light, which causes minimal gene expression change in *Prochlorococcus* (67), by using layered filters (#736 and #740 filters; LEE Filters, Burbank, CA) over a white light source. Axenic cultures were tested for purity by flow cytometry and by culturing into three broths: ProAC, ProMM, and MPTB (5, 66, 68).

### Flow cytometry and fast repetition rate fluorometry.

*Prochlorococcus* and A. macleodii MIT1002 cell abundances were measured on an Influx flow cytometer (Becton Dickinson, Franklin Lakes, NJ, USA), prepared and processed as previously described (69, 70). Calculations for relative chlorophyll per cell were created by normalizing red chlorophyll fluorescence per cell to 2-µm reference beads (catalog no. 18604; Polysciences, Warrington, PA, USA) as previously described (71). All flow cytometry files were analyzed using FlowJo version 7.6.5 (FloJo, LLC, Ashland, OR, USA).

Photosynthetic parameters were assessed daily using fast repetition rate fluorometry (FRRF) with a FIRe fluorometer system (Satlantic, Halifax, Nova Scotia, Canada). The maximum quantum efficiency of photochemistry in PSII (Fv/Fm) and the functional absorption cross section of PSII (σPSII) were measured and analyzed using Fireworx 1.0.4 (Audrey B. Barnett, Dalhousie University, Halifax, Nova Scotia, Canada), a collection of MATLAB functions to process FIRe fluorometry data.

### RNA extraction and RNA-seq library construction.

To extract total RNA, filters containing the cell biomass were first incubated in 10 mM Tris (pH 8), 20,000 U of Ready-Lyse lysozyme (Epicentre, Madison, WI, USA), and 40 U of SUPERase-In RNase inhibitor for 5 min at room temperature. RNA was then extracted using the mirVana microRNA (miRNA) extraction kit (Ambion, Carlsbad, CA, USA) according to the manufacturer’s instructions. All strand-specific transcriptome sequencing (RNA-seq) libraries were constructed identically, following a previously published protocol (16) that utilized a dUTP second-strand library construction approach (45) coupled with rRNA depletion by duplex-specific normalization (44). RNA-seq libraries for all replicate cultures at a given time point were constructed and sequenced simultaneously. Sequencing was carried out on an Illumina HiSeq2000 instrument at the MIT BioMicro Center, producing either 40-nucleotide (40-nt) single-end reads (axenic cultures) or 40-nt paired-end reads (cocultures). Paired-end reads were generated for coculture libraries to guarantee that all reads could be unambiguously assigned to the correct genome. One library which contained fewer than 300,000 total reads was excluded from analysis; all others yielded ~2.3 to 14.1 million reads (average, 5 million) per library (see Table S1 in the supplemental material).

### Transcriptome and statistical analysis.

Low-quality sequence regions were removed from the raw Illumina data using quality_trim (from the CLC Assembly Cell package, Qiagen/CLC Genomics, Aarhus, Denmark) with default settings (-c 20 –b 0.1 –l 0.5). The RNA-seq reads were aligned with the Burrows-Wheeler Aligner (72) to a reference file containing both the *Prochlorococcus* NATL2A and Alteromonas macleodii MIT1002 genomes (NCBI GenBank accession numbers CP000095.2 and JXRW01000000, respectively). The resultant alignments were parsed using the HTSeq package (73) using default settings to determine the number of reads aligned to each annotated ORF in the “sense” orientation. The abundance of rRNAs and tRNAs were not considered in the analysis due to the use of ribosomal depletion methods in preparing the libraries. Analyses of transcriptome composition counted a gene as being detected as long as there was at least one read mapped to a particular reading frame in the sense orientation in one of the replicate libraries from a given culture and time point.

Identification of significantly differentially abundant transcripts was performed with the DESeq2 R package (V1.10.0) (74), using standard DESeq2 functions and default workflows. Briefly, raw counts of reads mapping to each protein-encoding ORF (omitting rRNAs and tRNAs) across all time points and replicates were first compiled; reads mapping to the *Prochlorococcus* and A. macleodii MIT1002 genomes were stored in separate tables and analyzed independently. The standard DESeq2 pipeline was used to normalize differences in library sequencing depth and estimate gene-level dispersions using default options. We defined genes as having a high dispersion if their dispersion estimate was greater than 2. Tests for differential expression were performed between each pairwise condition of interest (i.e., the cocultured and axenic *Prochlorococcus* samples from a given time point) with the Wald test, using a negative binomial generalized linear model. The resulting *P* values were adjusted for multiple testing using the Benjamini-Hochberg procedure. Transcripts with an adjusted *P* value of <0.1 were considered to be significantly differentially abundant, as recommended by the DESeq2 authors (74).

Analysis of pathway-level changes in gene expression was carried out with PathwayTools V19.5 (75, 76), using the *Prochlorococcus* NATL2A Pathway/Genome Database (pmar59920cyc V19.0). Relevant lists of genes were imported as SmartTables in PathwayTools. Pathway enrichment/depletion was tested using default settings (Fisher exact test with a cutoff *P* of <0.1 [77]). Transcriptome correlation was calculated based on pairwise Spearman correlation coefficients of vectors containing the normalized, log-transformed read counts for all open reading frames for all unique pairwise combinations of biological replicates from a given time point. The number of genes exhibiting 24-h periodicity in expression was assessed with the R package “cycle” (78). As input to the periodicity analysis, we first applied the variance-stabilizing transformation, as implemented in DESeq2 (74), to all transcript count data between the 0- and 48-h time points. We then separated the 48-h time course data for the four different conditions (axenic versus cocultured, L:D cycle versus extended darkness) and averaged the normalized counts at each time point. This matrix was then used as input to the “cycle” package, specifying a cycle period of 24 h, the “ar1” background model, and *N* = 100; periodic expression was determined at a false-discovery rate of <0.10, as recommended by the authors (78).

All other statistical analyses were conducted within R (V 3.3.2) (79) or Microsoft Excel (Microsoft, Redmond, WA, USA) using the indicated test and multiple-testing correction as appropriate; significance was determined at a *P* value of 0.05. Figures were generated using ggplot2 (80).

### Accession number(s).

The data discussed in this publication have been deposited in NCBI’s Gene Expression Omnibus (81) and are accessible through GEO Series accession number GSE93197. All bacterial strains are available upon request.
