# Supplementary figures and images for: Heterotroph Interactions Alter Prochlorococcus Transcriptome Dynamics during Extended Periods of Darkness
Source: mSystems. 2018 May 29;3(3):e00040-18. doi: 10.1128/mSystems.00040-18 (PMC5974335; doi:10.1128/mSystems.00040-18)

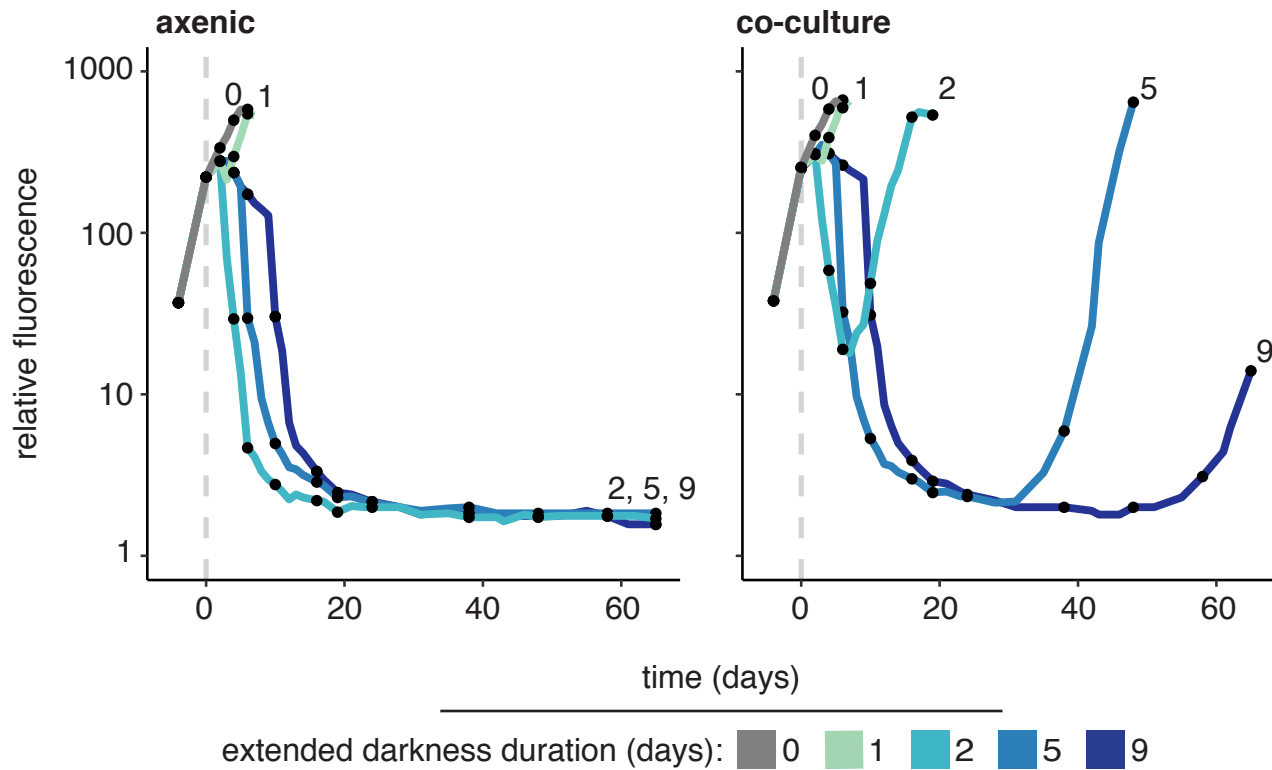

Supplement: FIG S1 [file sys003182233sf1.pdf]

## A 13:11 light:dark diel

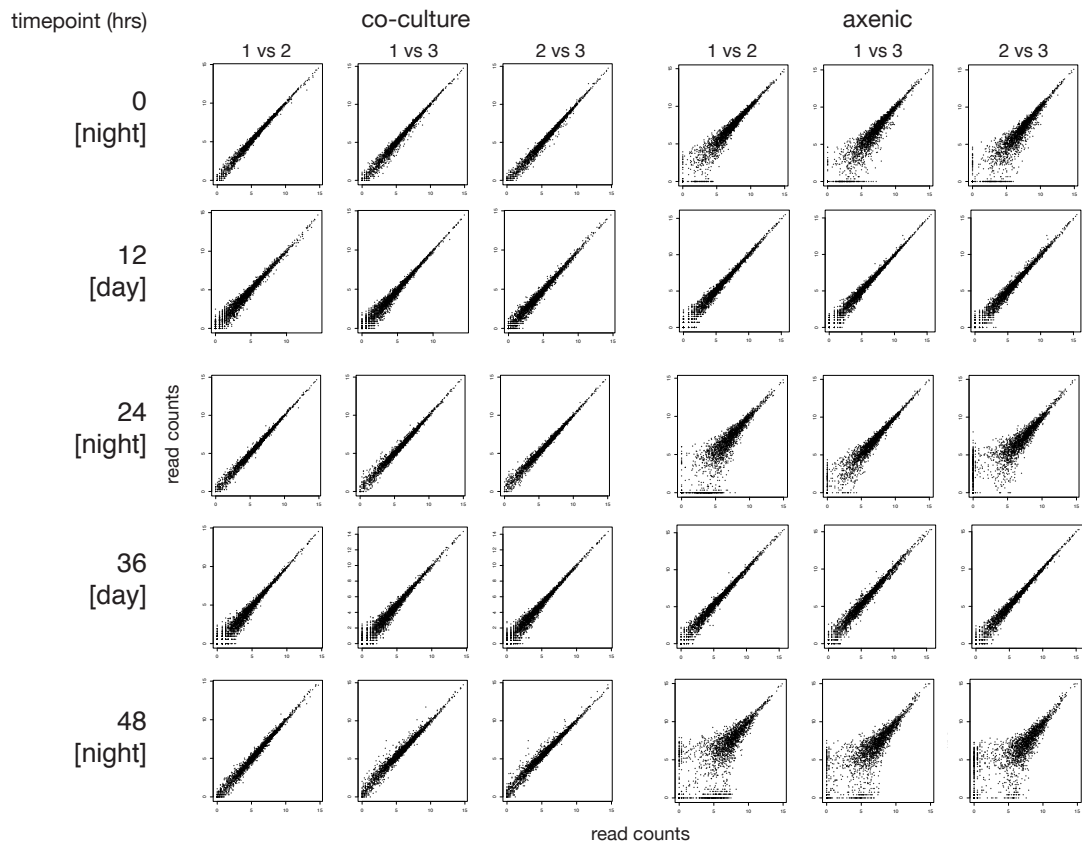

## B extended darkness

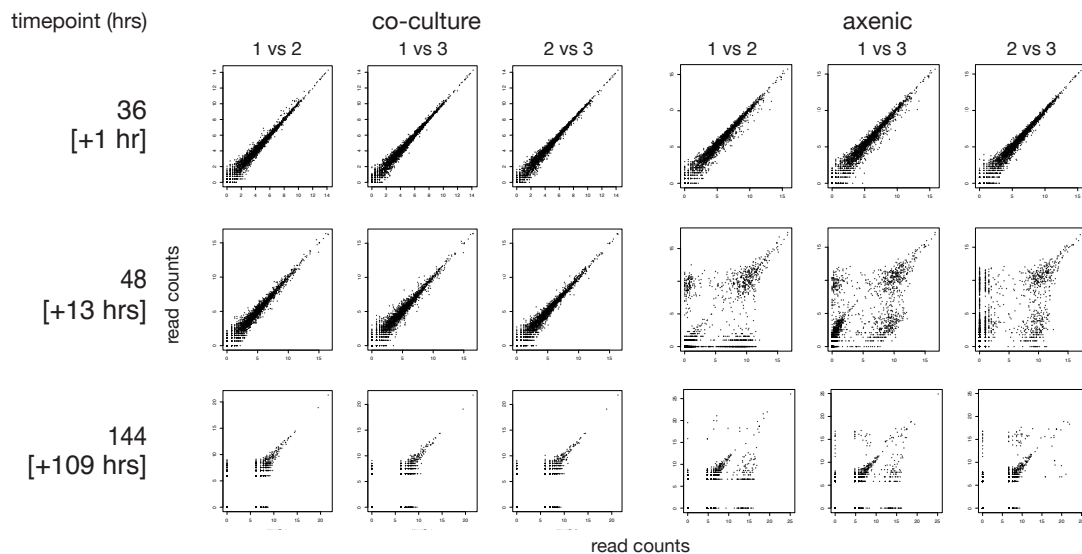

Supplement: FIG S2 [file sys003182233sf2.pdf]

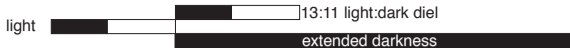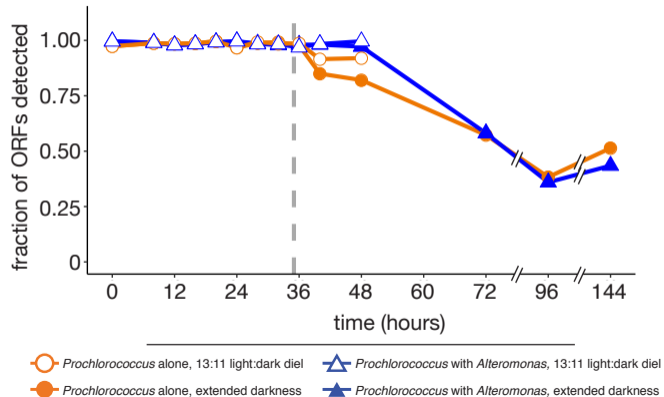

Supplement: FIG S4 [file sys003182233sf4.pdf]
