# Supplementary material for: Heterotroph Interactions Alter Prochlorococcus Transcriptome Dynamics during Extended Periods of Darkness
Source: mSystems. 2018 May 29;3(3):e00040-18. doi: 10.1128/mSystems.00040-18 (PMC5974335; doi:10.1128/mSystems.00040-18)
Supplement: FIG S3 [file sys003182233sf3.pdf]

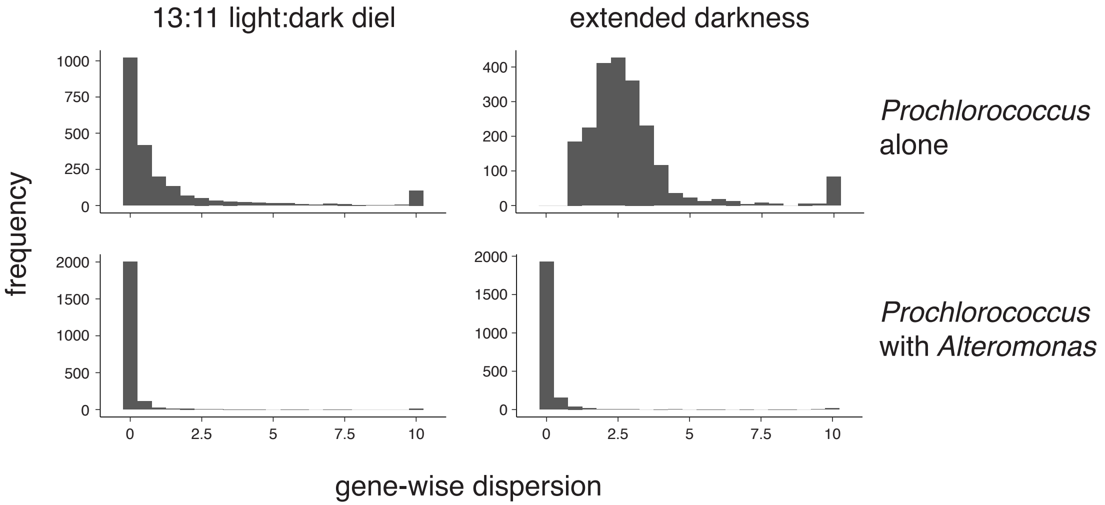

13:11 light:dark diel
extended darkness
Prochlorococcus alone
Prochlorococcus with *Alteromonas*
frequency
gene-wise dispersion
0
250
500
750
1000
100
200
300
400
1500
2000
2.5
5
7.5
10
